# Thermal Diffusivity of Aqueous Dispersions of Silicon Oxide Nanoparticles by Dual-Beam Thermal Lens Spectrometry

**DOI:** 10.3390/nano13061006

**Published:** 2023-03-10

**Authors:** Vladislav R. Khabibullin, Liliya O. Usoltseva, Ivan V. Mikheev, Mikhail A. Proskurnin

**Affiliations:** Analytical Chemistry Division, Chemistry Department, M.V. Lomonosov Moscow State University, d. 1, Str. 3, Lenin Hills, GSP-1 V-234, Moscow 119991, Russia; vladhab1995@gmail.com (V.R.K.); usoltsevalilya@gmail.com (L.O.U.)

**Keywords:** thermal lens spectrometry, thermal diffusivity, dispersions, thermal effects, measurement sensitivity

## Abstract

The growing interest in heat-conducting nanofluids requires highly sensitive methods for analyzing the thermal properties. Thermal lens spectrometry (TLS), despite its advantages over classical methods, does not have a general approach for measuring and interpreting results for dispersed systems. In this paper, for nanofluids of silicon oxide in water in a wide range of concentrations and sizes, the selection of measurement parameters for transient and steady-state thermal lensing is justified, and the interpretation of the results of thermal diffusivity measurements is substantiated. The features of the measurements of thermal diffusivity by TLS under stationary states for dispersed systems are considered. Using this approach, it is possible to detect and distinguish thermal effects with high accuracy. For dispersions of silicon oxide, with increasing concentrations, the thermal diffusivity passes through a minimum threshold. Silicon oxide dispersions can be used both as coolants or as heat-removing liquids by selecting the particle size and concentration.

## 1. Introduction

In many technological and scientific problems, heat-removing and cooling materials are required. To date, a large number of various heat-conducting materials have been proposed (glycerin, ethylene and propylene glycols, thermal pastes, freons, etc.), among which heat-conducting nanofluids as liquid coolants have gained particular recognition [1,2]. A nanofluid (NF) is a liquid suspension of nanoparticles (NPs) in common solvents such as water, ethylene glycol, etc. NF’s advantages result from the fact that solids have a high thermal conductivity (*k*) which contribute to the rapid heat transfer, but they also have a poor heat conservation due to the relatively low specific heat (*C_p_*). Liquids, on the contrary, due to their higher *C_p_*, are able to retain heat for a long time, but due to the lower *k*, they provide lower heat transfers [3]. Viscosity (*η*) is also a problem for heat transfer fluids, which limits the use of fluids and reduces the heat-transfer efficiency. The addition of NPs to a solvent contributes to a qualitative change in the heat-conducting and viscosity properties [4]. This makes the specific heat of a nanofluid somewhat lower than that of a pure solvent but leads to a substantial increase in the thermal conductivity of NFs when even small amounts of nanophase are added to the solvent [5,6].

The concentration and morphological features of the nanophase are crucial for the thermophysical properties of heat-conducting nanofluids. The need to measure thermophysical parameters is not limited to the measurement of *η* and *k*, and it is necessary to measure thermal diffusivity (*D*), which reflects the rate of the propagation/transfer of heat in the medium [7]. The thermal diffusivity of heat-conducting nanofluids depends on various factors: the nature of nanoparticles, their morphology, the nature of the solvent, pH, temperature, etc. [7], as well as evaluating the heat storage properties of heat-conducting nanofluids [6]. This requires accurate measurements and assessments of the thermal diffusivity.

However, there are difficulties in accurately determining thermal diffusivity and assessing the heat storage properties. Classical methods for measuring thermophysical parameters (3ω-method, non-stationary hot wire, heat flow, protected hot plate, etc.) [8] have significant disadvantages (thermal convection, low sensitivity to changes in the physicochemical composition, etc.), which make them unfitting for heat-conducting nanofluids. Against their background, photothermal lens spectrometry (thermal lens spectrometry, TLS) has some advantages for this task [9].

TLS is based on detecting changes in the refractive index. Under the action of radiation, molecules in the sample are excited. The absorbed energy is converted into thermal energy by nonradiative relaxation, which leads to the appearance of a temperature gradient, which usually acts as a diverging lens (thermal lens). As an optical method, TLS is highly sensitive to the physicochemical properties of liquid samples, which makes it possible to measure thermal diffusivity with high precision. Using TLS, it is possible to detect changes in *D* with low changes in the concentration (at mg to µg per mL range) [10,11,12] and NP morphology (size, layer thickness, particle shape) in nanofluids [13,14,15,16,17]. An important advantage of thermal lens spectrometry is its ability to detect the slightest convection processes in the solution. TLS quantitatively describes the Soret effect (thermophoresis) and thermal diffusion in dilute samples [9,18,19,20]. Here, the high sensitivity of the method makes it possible to study the heat transfer mechanism [11,21].

Still, TLS has some disadvantages that limit the method applicability, including work with optically transparent media and low absorbances [22], as well as a need for frequent adjustments of the spectrometer [23]. The impossibility of direct measurements of *C_p_* and *k* also limits the use of TLS [24].

By now, there are a few papers devoted to photothermal measurements of heat-conducting nanofluids mostly using transient variants of measurements [10,22,25,26]. The authors, as a rule, focused on solving narrow problems, limited to determining the *D* of specific nanofluids, the results of which cannot be extrapolated and applied to other systems [25,26]. In this respect, studies of the size effects of NPs are more universal and applicable to most systems [10]. For this reason, there is no general approach based on the often used Shen–Snook theory for homogeneous systems [22] to the analysis of thermal properties of heat-conducting nanofluids by TLS, since all NFs have different physicochemical properties. The problem is in modeling and selecting the optimum measurement conditions and interpreting the results.

In addition, a serious problem is the correctness of thermal lens measurements. As shown in [23], this problem is common to all measurements in TLS, and it is especially important for dispersed systems. Thermal effects (thermophoresis and convection) in an NF affect the development of the transient state of the thermal lens effect and the achievement of a steady state [18,27]. The problem is the lack of a suitable model for the measurement of transient curves that considers the contribution of thermal effects to the calculation of *D*. Because there are many non-equilibrium processes in disperse systems, it is impossible to use the entire transient curve to measure the thermal diffusivity.

Furthermore, in many studies, a reference is used for validation, which is a pure solvent with known optical and thermophysical parameters [12,28]. To confirm the correctness of the selected measurement parameters, the experimental results are approximated to the theoretical model and the thermal diffusivity of the found experimental one is compared with the theory (or according to reference data). In a few cases, when measuring reference samples, as we believe, there is a significant difference between the experimental and theoretical values of thermal diffusivity, which may affect the results of the research. Thus, in the study [11] the measured value of *D* for pure water was 0.182 × 10^−6^ m^2^/s (although, according to reference data, it is 0.143 × 10^−6^ m^2^/s [29]). Furthermore, the introduction of a correction factor for the measurement of the thermal diffusivity, as it is given in [21], in our opinion, can introduce an error in the reliability of the results.

In this paper, we consider the issues of measuring the thermal diffusivity by the dual-beam thermal lens spectrometry and the correct interpretation of the results. We compare results from TLS with the results obtained by the heat flow method. As an object of analysis, widely used and commercially available dispersions of Ludox silicon oxide nanoparticles are used in a wide range of concentrations and sizes. The results obtained complement the methodology for measuring heterogeneous systems by TLS. The presented approach to the description and interpretation of the results makes it possible to identify and describe various thermal effects in heterogeneous systems and thereby more clearly obtain the correct measurement results for the thermophysical parameters of dispersed systems.

## 2. Materials and Methods

### 2.1. Reagents and Chemicals

The study used dispersions of SiO_2_ nanoparticles of three different series of Ludox (SM-30, HS-40, TM-50) purchased from Merck (certificate descriptions are presented in Table 1). Aqueous dispersions were prepared using deionized water of the Milli-Q Reference system (Millipore SAS, Molsheim, France). We used the following solvents: ethanol (C_2_H_5_OH, CAS: 64-17-5, chemically pure, Khimreaktiv, Klin, Russia); chloroform (CHCl_3_, CAS: 67-66-3, analytical grade, Khimreaktiv, Klin, Russia); toluene (C_6_H_5_CH_3_, CAS: 108-88-3, analytical grade, LenReaktiv, St. Petersburg, Russia). Photostable indicator Ferroin (1,10-phenanthroline complex of iron(II), Fe(C_12_H_8_N_2_)_3_SO_4_, *M_r_* = 596.27 g/mol, CAS: 14634-91-4, analytical grade, LenReaktiv, St. Petersburg, Russia)) and dye Sudan 1 (C_16_H_12_N_2_O, *M_r_* = 248.28 g/mol, CAS: 842-07-9, chemically pure, Reakhim, Moscow, Russia) were used as chromophores. The mixing of solutions was carried out using a laboratory shaker model PE-6410 (EKROSKHIM, St. Petersburg, Russia).

For TLS, several samples of different brands were prepared with the same mass concentration of the solid phase of 1.60, 3.97, 8.79, 14.39, and 22.4 mg/mL (for TM series, 0.78, 2.80, 5.60, 11.2, and 16.8 mg/mL were additionally prepared). Ferroin was added to each sample. To reduce the systematic error caused by high absorbance, as described in [23], the concentration of ferroin in the dispersions did not exceed 1 µmol/L. After that, the dispersions were mixed for 2 h on a laboratory shaker and used in the analysis.

Based on the data in [23], we used highly diluted solutions as reference samples, which were a solution of ferroin in water with a concentration of 1 µmol/L and solutions of the dye Sudan 1 in chloroform, toluene, and ethanol with a concentration of 50 nmol/L.

### 2.2. Thermal Lens Measurements

We apply the Shen–Snook model for a dual-beam TLS with mode-mismatch in the stationary state [22]. We do not consider here the main conditions for the applicability of the model and briefly consider only the equations necessary to the work. The main equation for the intensity of the probe beam on the detector (transient curve equation) is:

(1) $$I\left(t\right)=I\left(0\right){\left[1-\frac{\theta }{2}\tan^{-1}\left(\frac{2mV}{\left[{\left(1+2m\right)}^{2}+V^{2}\right]\left(t_{c}/2t\right)+1+2m+V^{2}}\right)\right]}^{2},$$

in which the intensity of the probe beam $I\left(t\right)$ at each moment of the development of the thermal field (time *t*) depends on the characteristic time, $t_{c}$; the geometric parameters, *m* and *V*; and the thermo-optical signal, *θ* (*I*(0) is also present in the equation, the intensity of the probe beam at *t* = 0). The characteristic time, $t_{c}$, is related to thermal diffusivity, *D*, as:

(2) $$t_{c}=\omega_{e0}^{2}/4D$$

where $\omega_{e0}$ is the radius of the excitation beam in the waist. Thus, knowing the characteristic time and the excitation beam waist, we can find the thermal diffusivity of the analyte. However, to apply Equation (1), one must know three parameters: *m* is the mode mismatch factor, which is described by the equation:

(3) $$m={\left(\omega_{p1}/\omega_{e0}\right)}^{2}$$

containing the parameter representing the ratio of the radii of the probe ($\omega_{p1}$) and excitation beams at the center of the sample. *V* is the dimensional parameter of the spectrometer, which is described by the equation:

(4) $$V=z_{1}/z_{c}+z_{c}/z_{2}\left[1+{\left(z_{1}/z_{c}\right)}^{2}\right]$$

which takes into account the distance of the probe beam waist to the sample ($z_{1}$) from the sample to the detector ($z_{2})$ and the confocal distance (Rayleigh distance) for the probe beam $(z_{c}$), and the last parameter, *θ*, which includes the excitation beam power (*P*), linear absorption coefficient (*α*), optical path length (*l*), thermal conductivity (*k*), excitation laser wavelength (*λ_e_*), refractive index temperature coefficient (*dn*/*dT*):

(5) $$\theta =P\alpha l/\left(k\lambda_{e}\right)\cdot \left(-dn/dT\right)$$

In Equation (1), most parameters are constant and, for simplicity, it can be represented as:

(6) $$I\left(t\right)=I\left(0\right){\left[1-0.5\theta \tan^{-1}\left(a/\left(b\left(t_{c}/2t\right)+c\right)\right)\right]}^{2}\;$$

where *a*, *b* and *c* are constants: *a* = 2*mV*, *b* = (1 + 2*m*)^2^ + *V*^2^, *c* = 1 + 2*m* + *V*^2^.

The characteristic time from Equation (6) can be represented as a function of time:

(7) $$\tilde{t_{c}}\left(t\right)=\left[\left(a/\tan\;\left[2\cdot \left(1-\sqrt{I\left(t\right)/I\left(\infty \right)}\right)/\theta^{\prime }\right]\right)-c\right]\cdot 2t/b$$

where $\tilde{t_{c}}$(*t*) is the effective characteristic time at each point on the transient curve, and $\theta^{\prime }$ = 2[1−*I*(∞)/*I*(0)]/tan^−1^(*a*/*c*), where $\;I\left(\infty \right)$ is the probe laser intensity in the stationary state. In the case of a homogeneous solution, the steady state means the average value of the last points of the transient curve (in our case, the average of the last 300 ms). The thermal diffusivity, $\tilde{D}\left(t\right)$, the so-called effective thermal diffusivity, is calculated from Equation (7) for each value, $\tilde{t_{c}}\left(t\right),$ at time, *t*. The transition from effective $t_{c}$ and *D* to true values occurs by averaging the values of the first 100 ms of the functions $\tilde{t_{c}}\left(t\right)$ and $\tilde{D}\left(t\right)$ using Equation (2).

Before each measurement, we measured the characteristic time and the thermal diffusivity of the reference samples, which are pure solvents (with a small chromophore content) with precisely known thermophysical and optical parameters. All necessary constants in the thermo-optical parameter, *θ*, are taken from reference data and absorbance measurements.

We use the full development of the thermal field (a steady state). In this case, the thermal lens signal is:

(8) $$\vartheta =\frac{I\left(0\right)-I\left(\infty \right)}{I\left(0\right)}\;$$

Here, in the case of the homogeneous reference sample, we also use the average value of the last 300 ms of the transient curve as the steady state, but in the case of a heterogeneous system, we use the minimum value of *I*(*t*), which is the inflection point. For comparisons of the transient curves, we normalize them to the range of 0–1.

The development of the thermal lens signal, $\vartheta \left(t\right)$, was carried out using the calculation of the thermal lens signal at each point. Equation (8) was used for this, but $I\left(t\right)$ was used instead of $I\left(\infty \right)$, where $I\left(t\right)\;$ is the probe beam intensity at each time point. In addition, the development of the signal in time was normalized to the range of 0–1:

(9) $${\tilde{\vartheta }}^{\prime }\left(t\right)=\left[\vartheta \left(t\right)-\vartheta \left(0\right)]/[\vartheta^{\prime }\left(\infty \right)-\vartheta \left(0\right)\right]$$

where $\vartheta^{\prime }\left(\infty \right)$ is the signal in the equilibrium state, where the inflection point of a transient curve, $\vartheta \left(0\right),$ is the thermal lens signal at the initial moment of time.

To find the characteristic time and thermal diffusivity of heterogeneous systems, we used the stationary state of the transient curve. This means that, instead of $I\left(\infty \right)$, the corrected intensity of the probe beam, $I^{\prime }\left(\infty \right)$, is used, which is obtained by fitting the first 100 ms of the transient curve so that the last, 100th, point of the experiment is fully consistent with the theoretical one. In this case, the characteristic time can be found according to Equation (7) using the corrected intensity, $I^{\prime }\left(\infty \right)$:

(10) $${\tilde{t_{c}}}^{\prime }\left(t\right)=\left[\left(a/\tan\;\left[2\cdot \left(1-\sqrt{I\left(t\right)/I^{\prime }\left(\infty \right)}\right)/\theta^{\prime }\right]\right)-c\right]\cdot 2t/b.$$

The transition from effective characteristic times and thermal diffusivity ($t_{c}$’ and *D*’, respectively) to true ones was carried out in the same way as in the case of the homogeneous solutions described above, by averaging the data for the first 100 ms.

Transient curves are presented in three different normalized forms. In the first form, normalization is carried out on the largest value, $I\left(t\right)/I\left(0\right)$. In the second and third forms, they are normalized according to the following equation:

(11) $$\tilde{I}\left(t\right)=\left[I\left(t\right)-I\left(\infty \right)\right]/\left[I\left(0\right)-I\left(\infty \right)\right]$$

where $I\left(\infty \right)$ in the second form is the intensity of the probe beam in the stationary state, which is found by averaging the last 300 ms of the transient curve. In the third form, $I\left(\infty \right)$ is the intensity of the probe beam in the steady state (intensity value of the probe beam at ca. 100 ms). In this case, for the second form, we denote the normalized intensity of the probe beam by $\tilde{I}\left(t\right)$ as in Equation (11), without changes, and for the third form, ${\tilde{I}}^{\prime }\left(t\right)$ (with an apostrophe).

The transient curve for thermal lens dissipation,$\;I_{d}\left(t\right),$ was also normalized into two forms using the following equation:

(12) $${\tilde{I}}_{d}\left(t\right)=\left[I_{d}\left(t\right)-I_{d}\left(0\right)]/[I_{d}\left(\infty \right)-I_{d}\left(0\right)\right]$$

where $I_{d}\left(t\right)$ is the intensity of the probe beam at time *t*; $I_{d}\left(0\right)$ is the intensity of the probe beam at *t* = 0, at the moment the shutter closes, is the smallest value; $I_{d}\left(\infty \right)$ in the first form is the intensity of the probe beam at the stationary state, which is obtained by averaging the last 300 ms of the transient curve in the state of complete dissipation of the thermal lens and the return of the beam to the level of pure water. In the second form, the intensity of the probe beam is in the equilibrium state (intensity value at ca. 80–120 ms). In this case, for the first form, we denote the normalized intensity of the probe beam by ${\tilde{I}}_{d}\left(t\right)$ as in Equation (12), without changes, and for the second form, ${\tilde{I}}_{d}^{\prime }\left(t\right)$ (with an apostrophe).

The error is calculated according to the following equation:

(13) $$\Delta =\frac{X_{mes}-X_{true}}{X_{true}}\cdot 100\%,$$

where $X_{mes}$ is the measured value and $X_{true}$ is the true value calculated theoretically or from a reference.

### 2.3. Thermal Lens Spectrometer

**Figure 1:**
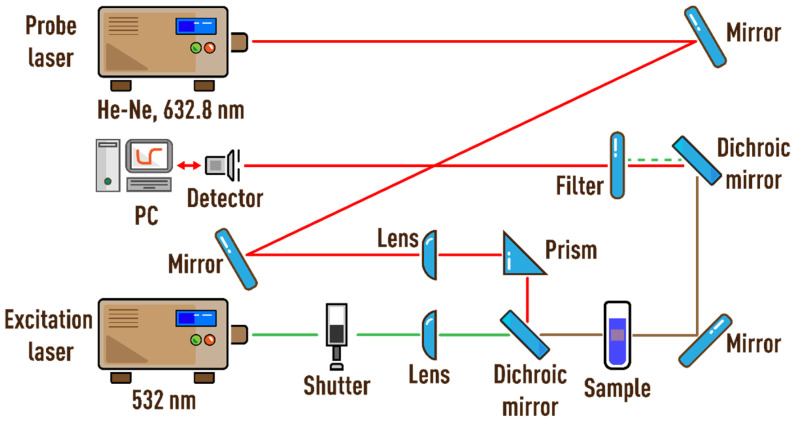
Schematic of the thermal lens setup.

The thermal lens spectrometer is described in detail in [23]. Figure 1 shows the scheme of the dual-beam thermal lens spectrometer. Radiation from the MGL-FN-532 solid-state laser (wavelength of 532 nm, TEM_00_; Changchun New Industries Optoelectronics Tech. Co., Ltd., Changchun, China) passes through a shutter (model SH05, ThorLabs, Newton, NJ, USA), which is controlled by an analog-to-digital and a digital-to-analog converter (ADC–DAC) model c8051Fx-DK (Silicon Labs, Boston, MA, USA) connected to a personal computer (PC), and enters the sample in a quartz cell (*l* = 10.00 mm), in which a thermal lens is generated. A helium–neon laser HNL050L (wavelength of 632.8 nm, TEM_00_; ThorLabs, Newton, NJ, USA,) was used as the probe laser. A photodiode was used as a detector.

The signal from the detector is recorded every cycle. One cycle starts when the shutter opens, continues when the shutter closes, and ends when the shutter is reopened, after which the cycle repeats. The PC receives data from the ADC and the detector, which are processed in the original program (C++ programming, Borland Corp., Austin, TX, USA), where the measurement cycles are formed, displayed, and stored in the form of transient curves (signal intensity vs. time). The laser power was measured using an Optronics Nova II power meter (Ophir Optronics Solutions, Jerusalem, Israel). The operating parameters of the measurements are summed up in Table 2.

The measurement parameters for the thermal lens spectrometer are optimized. The shutter frequency corresponds to the time of full development of the thermal lens and the time it takes for the transient curve to reach the steady state. The selection of the number of transient curves for averaging is based on the recommendations made in [23]. The mode-mismatch factor was selected based on the condition of the minimum systematic error identified in [23] and corresponding to the conditions of the model in [22]. The sample-to-detector distance also corresponds to the conditions of the model [22]. All systematic errors caused by the periodic divergence of the excitation beam, the relative displacement of two beams in the cell, the bias of the sample cell along the beam propagation, and the position of the maximum intensity of the probe beam in the center of the detector are taken into account and reduced to zero, according to the recommendations made in [23].

### 2.4. UV Visible Spectroscopy

For all samples, including reference samplers, spectra were recorded in the range of 350–700 nm to measure the absorbance (*A*) at λ = 532 nm. The spectra were recorded on a Cary 4000 spectrometer (Agilent Technologies, Santa Clara, CA, USA) using quartz cells with *l* = 10.00 mm.

### 2.5. Vibration Density Meter

The density of dispersions (ρ) was measured using a VIP-2MR vibration density meter for liquids (Thermex, Tomsk, Russia). The device is based on the standard test method ASTM 4052 and ASTM 5002.

### 2.6. Differential Scanning Calorimetry

The isobaric specific heat ($C_{p}$) of the dispersions was measured using the differential scanning calorimetry (DSC). Heat capacity measurements were carried out in a narrow temperature range of 15–40 °C. For this, a Mettler DSC 30 scanning calorimeter (Mettler Toledo, Toledo, OH, USA) was used. The specific heat of the samples was calculated from DSC data according to the ASTM E 1269-11 international standard.

### 2.7. Heat-Flow Method

Thermal conductivity (*k*) was measured by the heat-flow method using a FOX50 heat-flow meter (TA Instruments, New Castle, DE, USA). The instrument is based on the ASTM E1530 standard test method. The heat-flow meter directly determines the thermal conductivity value of the fluid. Thermal conductivity measurements were carried out in the temperature range of 10–70 °C with a difference of 20 °C between the heating and the heat-removing surfaces, as was conducted in [30].

The calculation of thermal diffusivity from the results of measurements by the heat-flow method was carried out according to the following equation:

(14) $$D=k/(C_{p}\cdot \rho ),\;$$

where k is the thermal conductivity measured by the heat-flow method, $C_{p}$ is the isobaric specific heat found by DSC, and ρ is the dispersion density measured on a vibrating densitometer.

## 3. Results and Discussion

In this paper, using a simple nanofluid with heat-conducting properties, we consider the use of thermal lens spectrometry for the analysis of thermophysical properties, with a model modified for dispersed systems. The paper substantiates the selection of measurement conditions and the processing of results. As an object of analysis, we used dispersions of Ludox silicon oxide nanoparticles, which have attractive physicochemical properties and are produced by a standardized technology. The dispersions are stable over a wide range of concentrations and can be diluted without a change in NP properties.

The results of the measurements of thermal diffusivity by TLS were compared with the heat-flow method. The latter is a widespread, simple, and highly reproducible method for measuring the thermophysical parameters of liquids and solid substances. The heat flow meter operates in a wide temperature range. The measurement error at room temperature for solids is about 3% [31]. The results of the heat flow method for nanodiamond aqueous dispersions were in good agreement with TLS [30].

The paper is structured in such a way that, first, the questions of the correctness of the thermal lens measurements are briefly considered using the example of homogeneous solutions. Then, the results of measurements of Ludox disperse systems are considered. Particular attention is paid to the features of the development and dissipation of the thermal lens and the advantages and disadvantages of various options for presenting the results, as well as questions of the correctness of processing the results. In conclusion, the features of the concentration dependences of the thermal diffusivity of various series of silicon oxides are considered, the results of TLS measurements are compared with the heat-flow method, and conclusions are made about the practical application of Ludox nanofluids.

### 3.1. Photothermal Measurements of Reference Samples

**Figure 2:**
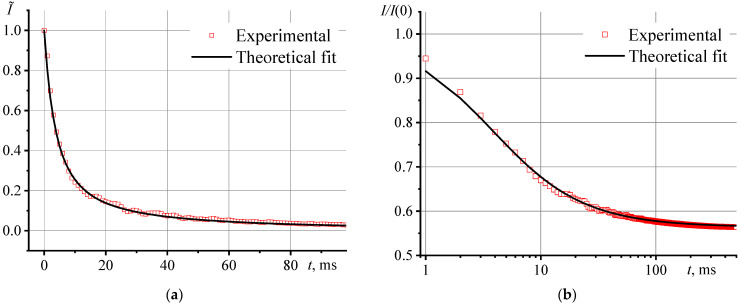
Transient curve for an aqueous solution of ferroin (1 µmol/L): (**a**) in linear scale form for the first 100 ms and (**b**) in logarithmic scale for the first 500 ms (measurement parameters are presented in Table 2).

To confirm the accuracy of the parameters and conditions for measuring the thermal diffusivity by TLS, before the analysis of aqueous dispersions of SiO_2_, we measured reference samples and solutions of chromophores in water, ethanol, chloroform, and toluene. In all cases, we observed the complete development of the thermal lens and the achievement of the steady state of transient curves, which indicates the absence of the Soret effect and thermal convection. The experimental transient curves are in good agreement with the model approximation without additional fitting. As an example, Figure 2a shows transient curves for an aqueous solution of ferroin in a normalized form for the first 100 ms. A plot in logarithmic coordinates (Figure 2b) shows the behavior of the transient curve more clearly than in a linear form.

The measurement of $t_{c}$ and *D* showed a good agreement with the reference data [32] for pure solvents (Table 3). The high accuracy and reliability of the experimentally found *t_c_* and *D* point to the optimum measurement conditions. Thus, we confirmed that the systematic error is negligible and that the TLS measurement parameters were selected correctly and optimally. All deviations from the theory, which will be observed for dispersions, we refer to only as deviations for a particular sample.

### 3.2. Photothermal Measurements of SiO_2_ Nanofluids

**Figure 3:**
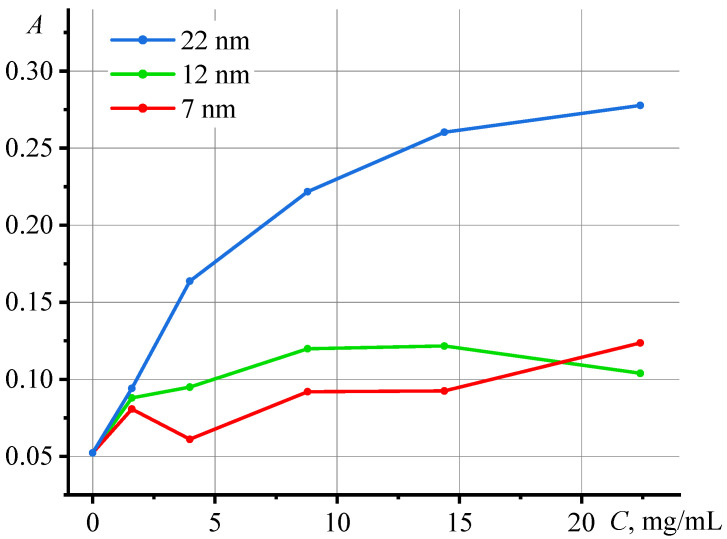
Dependence of absorbance at λ = 532 nm on the concentration of nanoparticles in Ludox dispersions.

The dispersions of silicon oxide nanoparticles in water are complex systems, in which several types of interactions with incident radiation occur. In addition to absorption, to a greater extent, light is significantly scattered. This affects sample optical properties due to the absence of a linear dependence *A* vs. *c* at the excitation laser wavelength of 532 nm (Figure 3). The UV-visible spectra of the dispersions are presented in the Supplementary Materials (Figure S1).

In TLS, the absorbed visible radiation transforms into thermal radiation and the heat propagates differently than it does in a true solution. These effects influence the development of the thermal lens and thermophysical properties. However, for all analyzed dispersions, similar dynamics of the development of transient curves was observed, which indicates the same thermal effects. For a simple presentation, we consider the results using certain variances as an example.

**Figure 4:**
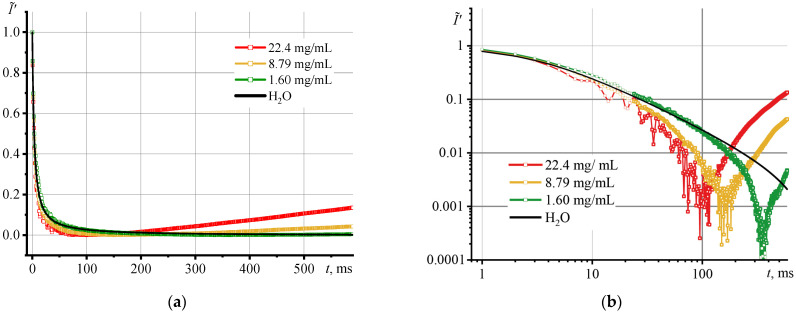
Transient curve, Equation (11), for dispersions of one series of NPs (HS, *d*_av_ = 12 nm), but at different concentrations for the first 600 s of thermal lens development, (**a**) in linear and (**b**) logarithmic scales.

Let us consider the results of thermal lens measurements of dispersions of SiO_2_ NPs with a medium particle diameter, 12 nm (HS series, Figure 4). With an increase in the concentration of the solid phase, the above effects become more pronounced. This is reflected in the appearance of a minimum value on the transient curve and in not reaching a stationary state. For the most concentrated dispersion (22.4 mg/mL, red line), the convection effects are significant compared to the diluted one (1.60 mg/mL, green line). The differences are most noticeable in logarithmic coordinates (Figure 4b). The thermal lens that appears upon the irradiation with an excitation laser develops faster in the most concentrated sample than in a blank reference solution, as evidenced by the larger initial slope of the experimental transient curve.

In ca. 100 ms, the trend of the transient curve changes and an increasing function appears, which indicates the predominance of thermophoresis (the Soret effect) due to overheating. Similar transient curves have been observed in studies of biodiesel, where such behavior was related to either the Soret effect or with photochemical or thermochemical processes [33,34]. However, in this case, there are no photochemical and thermochemical processes. A similar behavior of transient curves was demonstrated in [27]; the authors, using Ludox TMA series (*d*_av_ = 11 nm), observed a significant increase in the intensity after reaching a minimum. They recorded a decrease in the divergence of the probe beam because of the Soret effect for a dispersion with a mass fraction of only 2.5%. Thus, a converging lens appeared in the sample.

In the most diluted sample (1.60 mg/mL), a similar development of the thermal field is observed; the excitation laser causes overheating and the onset of thermophoresis (but much later than for the concentrated one). However, a closer look shows that up to ca. 80 ms, the transient curve passes over the curve for a reference solution, which indicates a slower growth of the thermal field in comparison with pure water, which shows a decrease in thermal diffusivity compared to pure water.

The Shen–Snook model describes the behavior of a thermal lens for dilute true solutions, where there are no optical distortions and thermophoresis [22]. In heterogeneous systems where thermal effects occur, the application of the Shen–Snook model without additional changes would lead to a high systematic error in the measurement of *D*. As a rule, the thermal diffusivity is found along the initial segment of the experimental transient curve by fitting the first 100–200 points to the model for homogeneous systems [35,36]. In our case, to describe transient curves, we applied normalization in several forms. To find *D*, we used the initial segment up to 100 ms.

Thus, using Equation (10), we can clearly distinguish between two different processes: thermal lens development (thus, measuring thermal diffusivity) and thermal diffusion (assessing the Soret effect). Nevertheless, we observed the almost complete independence of these processes for dispersions with low concentrations: the transient curve has reaches a steady state of the thermal lens, and then thermophoresis begins to develop. For dispersions with a higher concentration, the contribution of thermophoresis is larger, and development begins earlier. In this case, as can be seen from the transient curves for a diluted sample, the Soret effect is no more than 0.1%. Thus, we could distinguish and describe with high accuracy the thermal processes occurring in dispersions.

**Figure 5:**
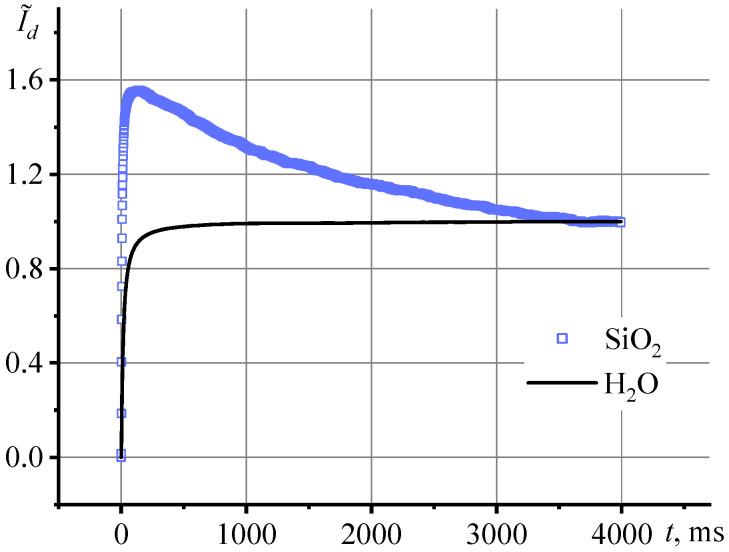
Transient curve for thermal lens dissipation, Equation (12), for a sample of Ludox (HS, *d*_av_ = 12 nm) with a concentration of 22.4 mg/mL.

Figure 5 shows the transient curves of the second part of the cycle for the HS-40 series with a concentration of 22.4 mg/mL. When the shutter is down and the excitation laser does not heat the sample, the dissipation of the thermal lens begins. This is an important part of the cycle where we observe the rate of sample cooling and the disappearance of the temperature gradient. Here, we observe a pronounced overheating of the dispersion, which manifests itself in the first ca. 200 ms of dissipation, followed by cooling to an ambient temperature at the end of the cycle. This behavior of the transient dissipation curve indicates heat-accumulating properties in the dispersion.

**Figure 6:**
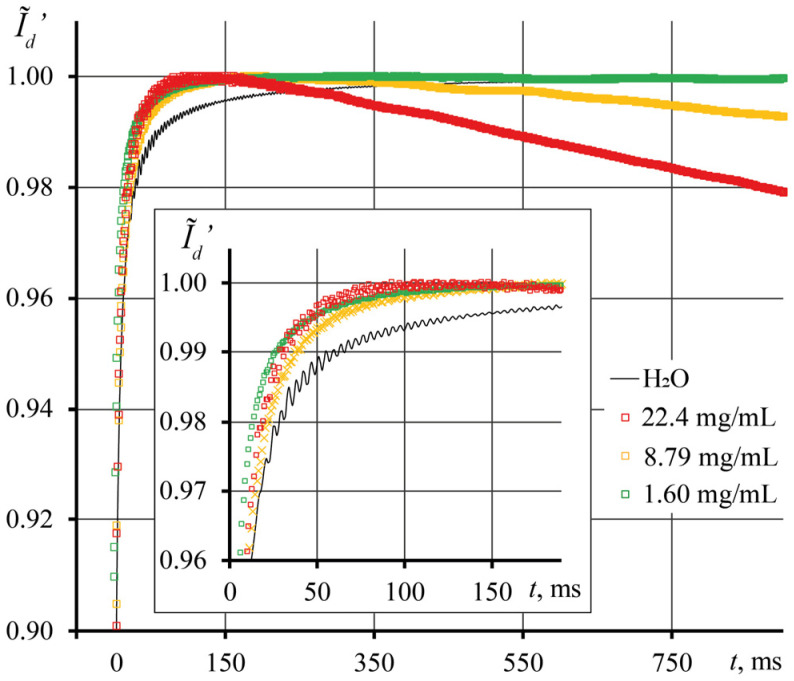
Transient curve for thermal lens dissipation, Equation (12), for samples of the same series (SM, *d*_av_ = 7 nm), but different concentrations for the first 900 s of dissipation (the first 200 ms in the inset).

For correct data interpretation, we present transient dissipation curves in a different form. Figure 6 shows the first 900 ms of cooling for a SM sample. With an increase in the concentration of the solid phase, the heat storage properties of the dispersion increase. However, for a correct description of the thermal lens, it is necessary to use the initial section of transient dissipation curves up to 200 ms (Figure 6, inset). This is since, after 200 ms, transient dissipation curves acquire a qualitative character, which describes not the dissipation of a thermal lens, but the slow cooling of the entire system due to the significant heat capacity. In Figure 6 (inset), we observe the cooling of the nanophase, which is faster than for pure water. In this case, the cooling rate increases with the concentration of NPs.

**Figure 7:**
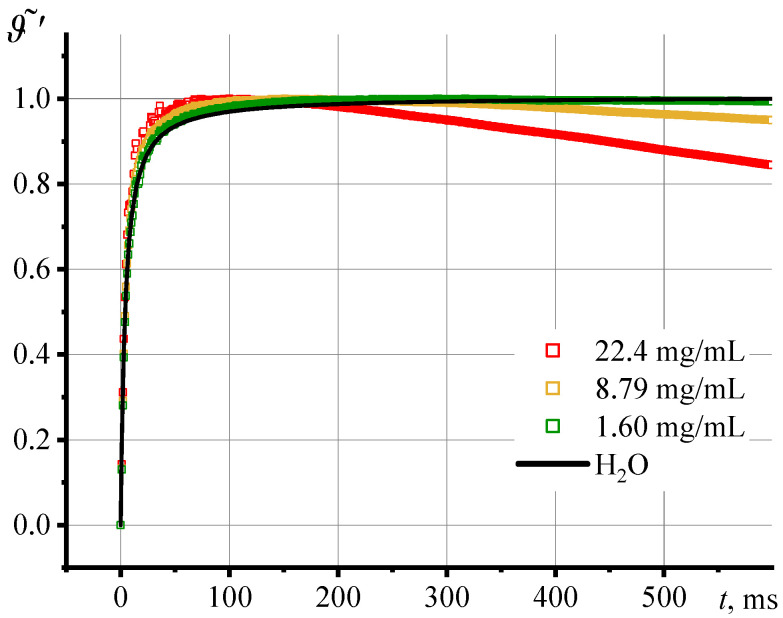
Transient thermal lens signal, Equation (9), for various concentrations of a dispersion of silicon oxide nanoparticles (HS series, *d*_av_ = 12 nm) for the first 600 ms.

If we consider the development of the thermal lens signal (Equation (9), Figure 7), similar dynamics are observed: all samples are characterized by an inflection of the *ϑ*(*t*) curve and the maximum value is reached at the point of inflection of *I*(*t*). Thus, the development of the thermal lens signal follows the dynamics of the transient dissipation curve.

### 3.3. Correctness of Thermal Lens Measurements

**Figure 8:**
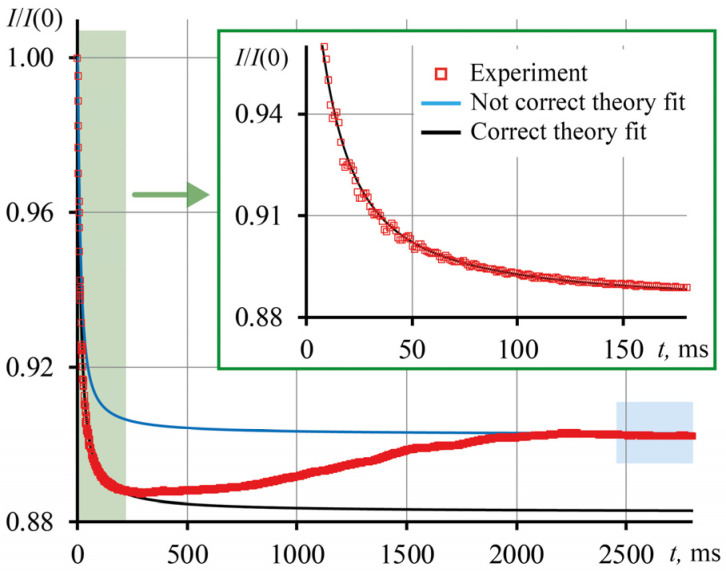
Transient curve for a HS sample (*d*_av_ = 12 nm, with a concentration of 22.4 mg/mL): red dots, experiment; the blue line is the theoretical transient curve constructed using the averaging of the last 300 points of the experimental transient curve as a steady state; the black line is the theoretical transient curve built by fitting the first 100 ms of the experiment.

The Shen–Snook model describes the development of the transient curve to a steady state well, but only in homogeneous, highly diluted solutions [22]. In dispersed systems, thermophoresis is present, and in extreme cases, thermal convection appears, which, with increasing concentration, makes a significant contribution to the transient curve and to the achievement of a steady state. In this case, the transient curve reaches a steady state, $I\left(\infty \right)$, which exceeds the true one (Figure 8, blue rectangle). The thermal lens signal calculated from $I\left(\infty \right)$ has an error, since it reflects the contribution of all thermal effects. The theoretical transient curve plotted using the probe-beam intensity steady state (Figure 8, blue line) cannot be used to find the characteristic time.

To find the thermal diffusivity of heterogeneous systems, as a rule, the approximation of the theoretical transient curve to the experimental one is used by fitting the parameters of Equation (1) so that the theory coincides with the experimental points of the transient curve (Figure 8, black line) [10,36,37]. In our case, we adjusted the intensities of the probe beam in the steady state, $I^{\prime }\left(\infty \right)$, in such a way that the first 100 ms of the development of the experimental transient curve would be compared with the theoretical one, since we consider this area most informative (Figure 8, blue rectangle). Table 4 presents the results of measuring the characteristic time and thermal diffusivity for the SM series (average particle diameter, 7 nm) using thermal lens steady states: apparent in Equation (7) and apparent-corrected in Equation (10). The largest error in the measurement of the characteristic time, as expected, is achieved at the highest concentration of particles, where the thermal and optical effects manifest themselves to a larger extent.

### 3.4. Thermal Diffusivity of SiO_2_ Nanofluids

**Figure 9:**
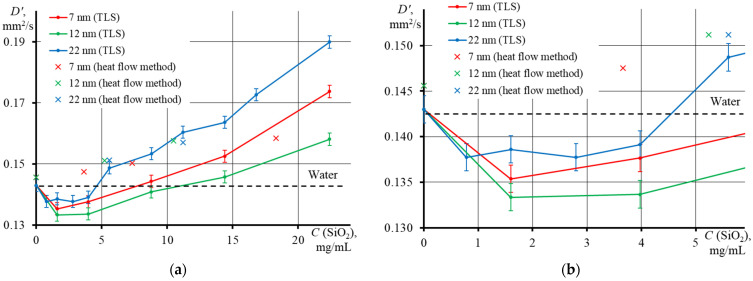
Thermal diffusivity of dispersions of various Ludox series (SM, *d_av_* = 7 nm; HS, *d_av_* = 12 nm; TM, *d_av_* = 22 nm) where: (**a**) in the concentration range from 0 to 22.4 mg/mL, (**b**) up to 5 mg/mL (*n* = 5, *p* = 0.95). The crosses are measurements by the heat flow method, Equation (14), and the lines are measurements by thermal lens spectrometry, Equations (10) and (2).

Figure 9 shows the results of measuring *D* by two methods: TLS (round dots and lines) and the heat-flow method (crosses). Thermal diffusivity measured by the heat-flow method (Figure 9, crosses) for all types of dispersions and concentrations differs slightly from each other for low concentrations (Figure 9b). This may be due to thermal convection and different sample conditions [31,38]. Our results confirm the low sensitivity of the heat-flow method at low concentrations and morphological features of dispersions. It should be noted that the heat-flow method is mainly used for solids in which thermal equilibrium is reached quickly, and the error in determining the thermal conductivity at room temperature does not exceed 3% [31].

TLS, on the contrary, show evident differences in thermal diffusivity depending on the NP dispersion series. In addition, at low concentrations, up to 5 mg/mL (Figure 9b), a decrease in thermal diffusivity was found for all particle sizes. In this range, the influence of the thermal conductivity of the system predominates. This behavior was previously found on finely dispersed systems of quantum and carbon dots, nanoparticles, etc. [10,14,35]. The authors of these studies explain this by the fact that the chromophore molecules, upon absorbing radiation, scatter it into the environment in the form of heat. Nanoparticles absorb heat from molecules and transfer it to the environment. At the same time, the Brownian motion is considered to be the key mechanism for heat transfer: the higher the concentration, the more difficult it is for heat transfer to neighboring NPs and the lower the thermal diffusivity [35]. Previously, with an aqueous dispersion of ZnO–graphene oxide composites as an example, it was found that an increase in *D* is observed at low concentrations, and a sharp decrease was found at higher concentrations in [11]. This allowed the authors to make assumptions about the heat transfer mechanism. They associated it with the Brownian motion of NPs in the medium and the leading role of nanoparticles in the composite for heat transfer: when the concentration is low, Brownian motion is high and heat is transferred quickly, but when a certain concentration is reached, composites agglomerate, which prevents the free movement of NPs in the volume and the thermal conductivity decreases [11].

The second segment of the curve, the range of concentrations >5 mg/mL, shows a linear increase in the heat transfer rate with the concentration of NPs. In this case, the heat storage properties increase and the increase in the heat capacity of the system predominates. This behavior was found in [39] and can be explained as follows: with an increase in the average particle size, the total surface area decreases, and, with it, the solid/liquid base interface area decreases, which leads to a decrease in the interfacial thermal resistance and an increase in the thermal conductivity of the nanofluid [7,40]. An increase in *D* was observed not only with the addition of small amounts (µg and mg) of NPs to pure solvents (water, ethanol, and ethylene glycol) [26,37], but also with an increase in the nanophase concentration. This has been observed in acrylic resin [25], water [41], ethanol, and ethylene glycol [37].

However, the rate of change in *D*, at first glance, does not have a strict dependence on the particle size. The largest growth is observed in the sample with the largest average particle diameter (22 nm, TM series), and the smallest growth is observed in NPs with *d*_av_ = 12 nm (HS series). At the same time, SiO_2_ with *d*_av_ = 7 nm (SM series) showed an intermediate value. Previously, it was found that the smaller the particle size, the more the thermal diffusivity decreases [36]. The authors also associated this behavior with the Brownian motion in the following way: if the particle size is very small, the Brownian motion is significant and the heat transfer rate is high, and if the particles are large, then the Brownian motion is slight and heat propagation is difficult.

**Figure 10:**
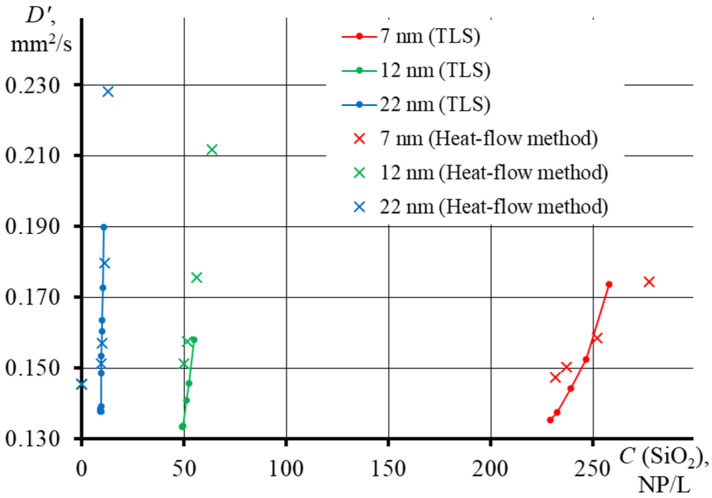
Effect of particle concentration on thermal diffusivity of dispersions for different Ludox series (SM, *d*_av_ = 7 nm; HS, *d*_av_ = 12 nm; TM, *d*_av_ = 22 nm), where crosses are heat flow measurements, Equation (14) and lines are TLS measurements, Equations (10) and (2).

Let us consider the dependence of *D* on particle concentration, accounting for the average particle diameter and surface area (Figure 10). It was found that the smallest contribution to the thermal conductivity is rendered by the particles with the smallest diameter, where the largest concentration is required for a noticeable change in *D*, while the lowest concentration is required for the largest particles (thermal diffusivity graphs for individual Ludox series are presented in the Supplementary Materials, Figure S2). This is in good agreement with the above concept of the heat transfer mechanism and with the results obtained previously. Studies of heat-conducting nanofluids with ferrimagnetic [13] and silver nanoparticles [42,43] have shown that, with an increase in the NP size, thermal conductivity increases. The heating rate, therefore, will be the highest for large particles and the lowest for small ones. This indicates that when measuring thermal diffusivity for disperse systems, it is necessary to make a correction with due account for the morphological properties of the nanophase. However, based on Figure 10 and Figure S2, we also confirmed that the heat-flow method has a poor sensitivity to the nanoparticle size.

Previous studies on the influence of the shape of nanoparticles on thermal diffusivity by the TLS showed that the NPs of rod shapes improve heat diffusion in a liquid more efficiently than spherical ones [44]. Using Pt–Au bimetallic particles, the influence of the NP composition on the thermal diffusivity of a nanofluid was established [45]. The dependence of *D* on the thickness of the gold shell on silver nuclei was revealed, and it was found that with an increase in the coating thickness, the thermal diffusivity of aqueous dispersions increases [28].

**Figure 11:**
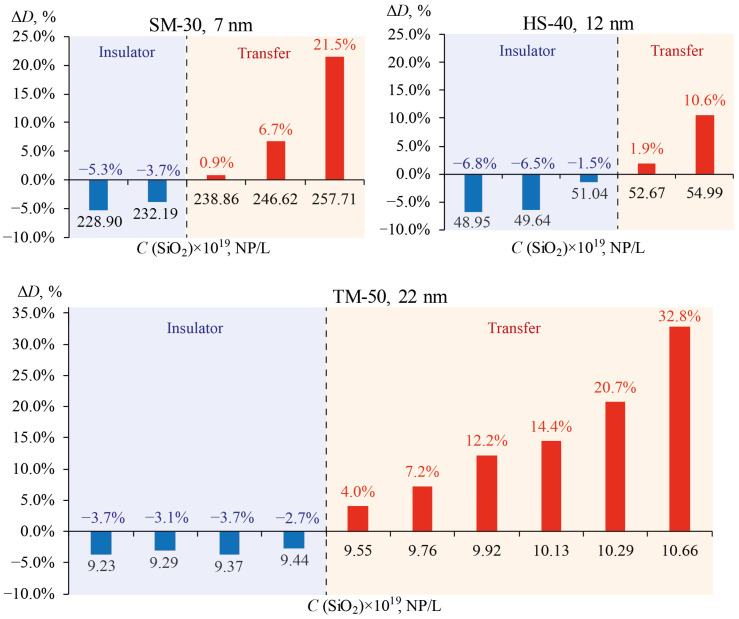
Increase in thermal conductivity for SiO_2_ dispersions of various Ludox series in comparison with pure water. A decrease is denoted with negative values (blue color), and an increase is denoted with positive values (red color).

Below are plots of the changes in thermal diffusivity for all the studied particles of relatively pure water (Δ*D*, Figure 11), which are consistent with the results above. The largest increase in *D* is observed for particles with the largest diameter, and the smallest increase for particles with the smallest diameter. The thermal diffusivity of nanofluids increases with an increase in the optical absorption of nanoparticles [11]. For NPs with high optical absorptions (quantum dots, metal NPs, etc.), a significant change in the thermal diffusivity is observed at a concentration of 0.01–0.1 mg/mL [14,17]. In our case, even though silicon oxide nanoparticles weakly absorb radiation, it is clearly shown that a significant change in *D* occurs at concentrations above 5 mg/mL. In the region of low concentrations (up to 5 mg/mL), where a drop in thermal diffusivity is observed, the dispersions exhibited the properties of a heat insulator. Similar dynamics were observed for quantum dots, where at a concentration of 0.05 mg/mL, the decrease in *D* was at a maximum and amounted to more than 60% [14]. The region of higher concentrations, where the thermal diffusivity of dispersions increases, is suitable for the purposes of heat transfer and cooling [3]. The greatest increase in thermal diffusivity, relative to pure water, was observed in the largest particles, where Δ*D* was more than 30%, at a concentration of 22.4 mg/mL. For example, in [4], the increase in thermal diffusivity for an aqueous dispersion of carbon nanotubes with a concentration of 20 mg/mL was no more than 10%. Thus, to obtain a coolant with a greater efficiency, particles with the largest diameter are suitable because they require a smaller amount of solution than smaller ones.

Thus, the results obtained in this paper are in good agreement with the literature data. Silicon oxide dispersions can be used both as coolants and as heat-removing liquids by adjusting the particle size or concentration.

The following are general recommendations for measuring the thermal diffusivity of disperse systems using double-beam thermal lens spectrometry: (i) before analyzing disperse systems, it is necessary to optimize the selection of the measurement parameters using a standard, (ii) measure the thermal diffusivity using the data for first 100–150 ms of the development of the transient curve, (iii) consider the morphological features of the dispersions when describing the results.

## 4. Conclusions

Thermal diffusivity is an important parameter for heat-conducting nanofluids. The work with low concentrations of the nanophase in heat-conducting nanofluids requires sensitive measurement methods. This paper presents an approach to the analysis of nanofluids by thermal lens spectrometry. For this, the Shen–Snook model was adapted to measure the thermal diffusivity of dispersed systems. The thermal conductivity found by thermal lens spectrometry was compared with the heat flow method. As the results showed, the heat-flow method was unable to reliably characterize systems in the range of up to 10–15 mg/mL, as well as to reveal the differences in the morphological features of the particles in the dispersion. On the other hand, thermal lens spectrometry has been found to have a higher sensitivity and perform well in these tasks. For Ludox SiO_2_ nanofluids, at low concentrations (up to 5 mg/mL), a decrease in thermal diffusivity was revealed, due to the contribution of thermal conductivity. Further, with increasing concentration, the contribution of the heat capacity begins to dominate and the thermal diffusivity increases. Using thermal lens spectrometry, it was also found that with a change in the particle size, the thermal diffusivity also changes. Nevertheless, for the correct presentation of the results of the measurements of the thermal diffusivity of dispersions, it is necessary to consider the nanophase size. TLS revealed the heat-accumulating properties of dispersions and differences in thermal effects, which is also an important thermophysical problem. Thus, thermal lens spectrometry is a versatile tool in the analysis of thermal diffusivity in the range of low concentrations, which classical methods are unable to solve.

## Figures and Tables

**Table 1:** Parameters of Ludox dispersions.

Ludox	Average Particle Diameter *d*_av_, nm	Concentration, % *w*/*w*	Density, kg/m^3^	Specific Surface, m^2^/g
SM-30	7	30	1.220	350
HS-40	12	40	1.310	220
TM-50	22	50	1.400	140

**Table 2:** Thermal lens measurement parameters.

Parameter	Value
Excitation laser	
Wavelength, *λ_e_* (nm)	532
Focusing lens focal length, *f_e_* (mm)	200
Confocal distance, *Z_ce_* (mm)	10.9
Laser power at cell, *P* (mW)	120
Spot size at the waist, *ω_e_*_0_ (µm)	42
Probe laser	
Wavelength, *λ_p_* (nm)	632.8
Focusing lens focal length, *f_p_* (mm)	300
Confocal distance, *Z_cp_* (mm)	2.7
Laser power at cell (mW)	4.5
Spot size at the waist, *ω_p_*_0_ (µm)	23
Spot size at cell, *ω_p_* (µm)	90
Other constants	
Cell length (mm)	10
Sample-to-detector distance, *Z*_2_ (cm)	230
Mode mismatch factor, *m*	4.59
Geometric parameters, *V*	4.89
Modulator frequency (Hz)	0.25
Number of transient curves to average	300
Number of experiment repetitions	5

**Table 3:** Thermophysical parameters of reference samples (*n* = 5, *p* = 0.95).

Solvent	Characteristic Time, ms			Thermal Diffusivity, mm^2^/s		
	Calculation, Equation (2)	Experiment, Equation (7)	Δ, %	Theory [32]	Experiment	Δ, %
Water	3.10	3.04 ± 0.04	2	0.142	0.145 ± 0.002	2
Ethanol	4.95	4.95 ± 0.03	<1	0.089	0.089 ± 0.001	<1
Chloroform	5.44	5.44 ± 0.10	<1	0.081	0.081 ± 0.002	<1
Toluene	4.79	4.85 ± 0.23	1	0.092	0.091 ± 0.005	1

**Table 4:** Error in finding the characteristic time and thermal diffusivity, Equation (13), between using the averaging of the last 300 ms of the transient curve as a stationary state (parameters without apostrophe) and using the adjusted intensities of the probe beam in the stationary state (parameters with apostrophe, true value).

Ludox	*c*, mg/mL	Not Correct, Equation (7)		Correct, Equation (10)		Δ(*D*), %
		*t_c_*, ms	*D*, mm^2^/s	*t_c_′*, ms	*D′*, mm^2^/s	
SM (*d*_av_ = 7 nm)	1.60	2.23	0.198	3.32	0.133 ± 0.002	49
	3.98	1.48	0.298	3.24	0.136 ± 0.001	120
	8.79	0.38	1.161	3.20	0.138 ± 0.002	740
	14.39	0.20	2.205	2.88	0.153 ± 0.002	1340
	22.40	0.12	3.675	2.53	0.174 ± 0.004	2010

## Data Availability

Not applicable.

## Supplementary Materials

The following supporting information can be downloaded at: https://www.mdpi.com/article/10.3390/nano13061006/s1. Figure S1: UV-visible spectra for Ludox with different concentrations of the solid phase: (a) SM with *d*_av_ = 7 nm, (b) HS with *d*_av_ = 12 nm, (c) TM with *d*_av_ = 22 nm; Figure S2: Thermal diffusivity of various Ludox with different concentrations of the solid phase measured by the TLS (Equations (10) and (2); lines), and heat-flow method (Equation (14); crosses): (a) SM with *d*_av_ = 7 nm, (b) HS with *d*_av_ = 12 nm, (c) TM with *d*_av_ = 22 nm.
