# Supplementary material for: Thermal Diffusivity of Aqueous Dispersions of Silicon Oxide Nanoparticles by Dual-Beam Thermal Lens Spectrometry
Source: Nanomaterials (Basel). 2023 Mar 10;13(6):1006. doi: 10.3390/nano13061006 (PMC10056864; doi:10.3390/nano13061006)
Supplement: Supplementary file 1 [file nanomaterials-13-01006-s001.zip › nanomaterials-2258934-supplementary.pdf]

Figure

Figure S1. UV-visible spectra for Ludox with different concentrations of the solid phase: (a) SM with d_{av} = 7 nm, (b) HS with d_{av} = 12 nm, (c) TM with d_{av} = 22 nm;

Figure S2. Thermal diffusivity of various Ludox with different concentrations of the solid phase measured by the TLS (Equations 10 and 2; lines,) and heat-flow method (Equations 14; crosses): (a) SM with d_{av} = 7 nm, (b) HS with d_{av} = 12 nm, (c) TM with d_{av} = 22 nm.

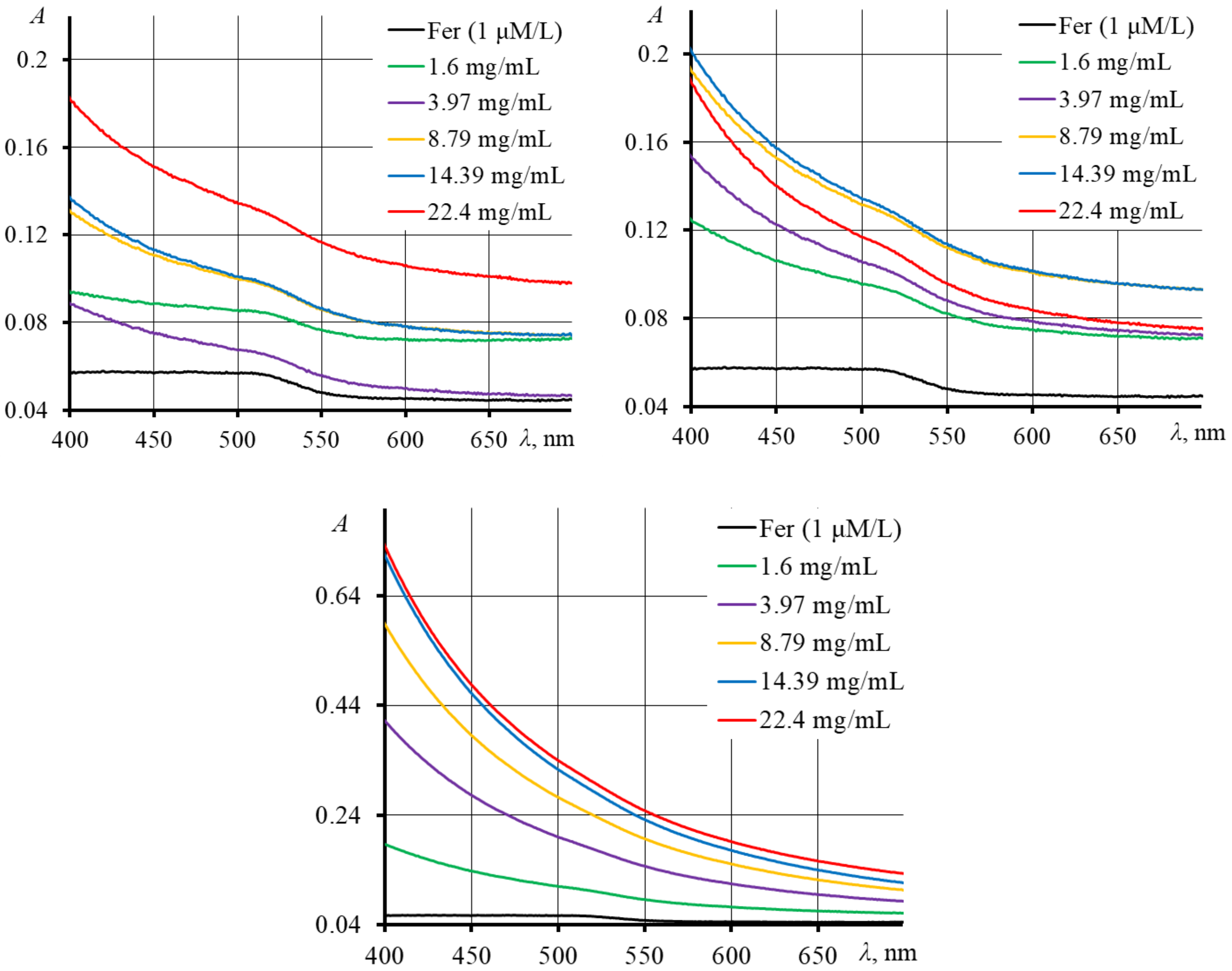

Figure S1. UV-visible spectra for Ludox with different concentrations of the solid phase: (a) SM with d_{av} = 7 nm, (b) HS with d_{av} = 12 nm, (c) TM with d_{av} = 22 nm;

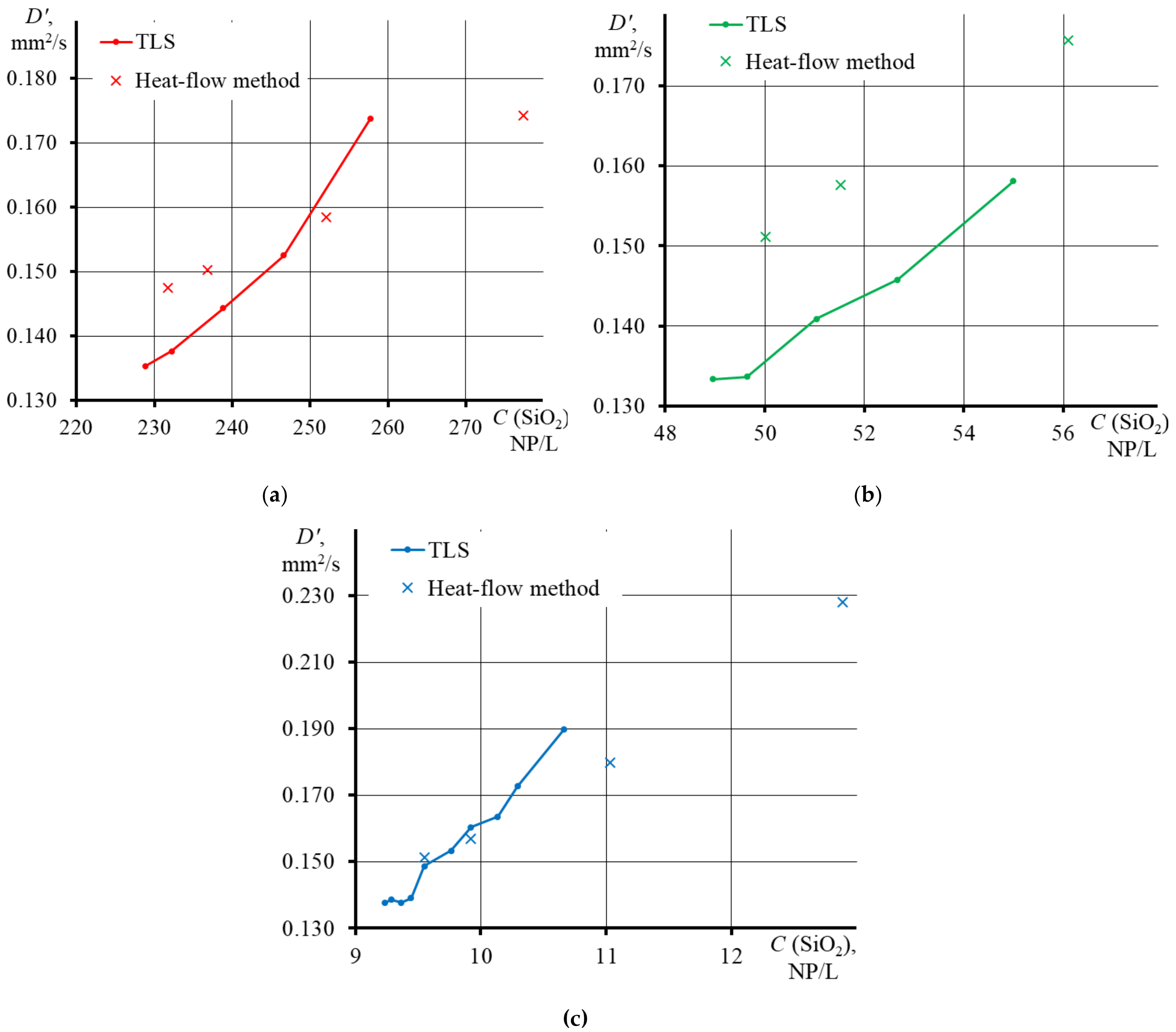

Figure S2. Thermal diffusivity of various Ludox with different concentrations of the solid phase measured by the TLS (Equations 10 and 2; lines,) and heat-flow method (Equations 14; crosses): (a) SM with d_{av} = 7 nm, (b) HS with d_{av} = 12 nm, (c) TM with d_{av} = 22 nm.
